# Pan‐cancer analysis revealing DAAM1 as a novel predictive biomarker for PD‐1/PD‐L1 blockade in clear cell renal cell carcinoma

**DOI:** 10.1002/mco2.177

**Published:** 2022-10-27

**Authors:** Jie Mei, Honghong Fan, Jiaofeng Zhou, Dingwei Huang, Junying Xu, Yichao Zhu

**Affiliations:** ^1^ Department of Oncology The Affiliated Wuxi People's Hospital of Nanjing Medical University Wuxi Jiangsu China; ^2^ Department of Physiology Nanjing Medical University Nanjing China

Dear Editor

Disheveled‐associated activator of morphogenesis 1 (DAAM1) is a member of Formin protein family, which binds to the growing barbed ends and mediates microfilament polymerization during the formation of cell pseudopodia.^1^ DAAM1 is evolutionarily conserved and contains two highly homologous domains, Formin homology domains 1 and 2 (FH1 and FH2).^2^ Emerging evidence has uncovered that DAAM1 functions as a critical oncogene in facilitating tumor metastasis of multiple cancers. Our previous research revealed that DAAM1 was highly expressed in breast cancer tissues and was mediated by YWHAZ protein, and the cooperation between YWHAZ and DAAM1 contributed to breast cancer metastasis.^3^ In addition, DAAM1 was also essential for metastasis and invasion of lung cancer.^4^ However, the systematic analysis of the role of DAAM1 in pan‐cancer has not been observed. In the current research, we analyzed the expressions and prognostic values of DAAM1 across cancer types and focused on the immuno‐correlation of DAAM1 in kidney renal clear cell carcinoma (KIRC).

Firstly, we explored the levels of DAAM1 mRNA expression in multiple tumor types using the RNA‐sequencing data from The Cancer Genome Atlas (TCGA) dataset. The results showed that DAAM1 was upregulated in glioblastoma multiforme (GBM), lower grade glioma (LGG), prostate adenocarcinoma (PRAD), stomach adenocarcinoma (STAD), pancreatic adenocarcinoma (PAAD), and cholangio carcinoma (CHOL), and downregulated in uterine corpus endometrial carcinoma (UCEC), lung adenocarcinoma (LUAD), esophageal carcinoma (ESCA), kidney renal papillary cell carcinoma (KIRP), colon adenocarcinoma (COAD), KIRC, lung squamous cell carcinoma (LUSC), skin cutaneous melanoma (SKCM), bladder urothelial carcinoma (BLCA), rectum adenocarcinoma (READ), ovarian serous cystadenocarcinoma (OV), testicular germ cell tumor (TGCT), uterine carcinosarcoma (UCS), adrenocortical carcinoma (ACC), and kidney chromophobe carcinoma (KICH) (Figure S1A). With regard to the prognostic values of DAAM1 in different cancers, we found that they were not significant in most cancer types. However, high DAAM1 expression was associated with well prognosis in KIRC, which was inconsistent with its oncogenic roles in most cancers (Figure S1B–D). Thus, we focused on the role of DAAM1 in KIRC in subsequent analysis.

Next, we deeply investigated the expression and prognostic value of DAAM1 in KIRC. We found that DAAM1 protein expression detected by immunohistochemistry staining was significantly downregulated in three independently validated cohorts (Figures 1A and S2A,B). In addition, to determine the best cut‐off value of DAAM1 expression in predicting prognosis, we divided KIRC patients from the TCGA cohorts into three equal groups according to the expression of DAAM1. We found that the prognosis of patients with high and medium DAAM1 expressions was similar, but those with low DAAM1 expression was significantly poor (Figure S3A–C). Thus, KIRC patients with DAAM1 expression at one‐third from the bottom were regarded as the low expression group, and others were regarded as the high expression group (Figure S3D–F). Furthermore, in the validated cohorts, DAAM1 expression was significantly associated with survival status and high DAAM1 expression predicted well prognosis (Figure 1B,C). In addition, univariate and multivariate analyses indicated that DAAM1 was an independent survival‐related factor in KIRC (Table S1). Overall, high DAAM1 expression was associated with well prognosis in KIRC and could be as an independent survival‐related factor.

**FIGURE 1:**
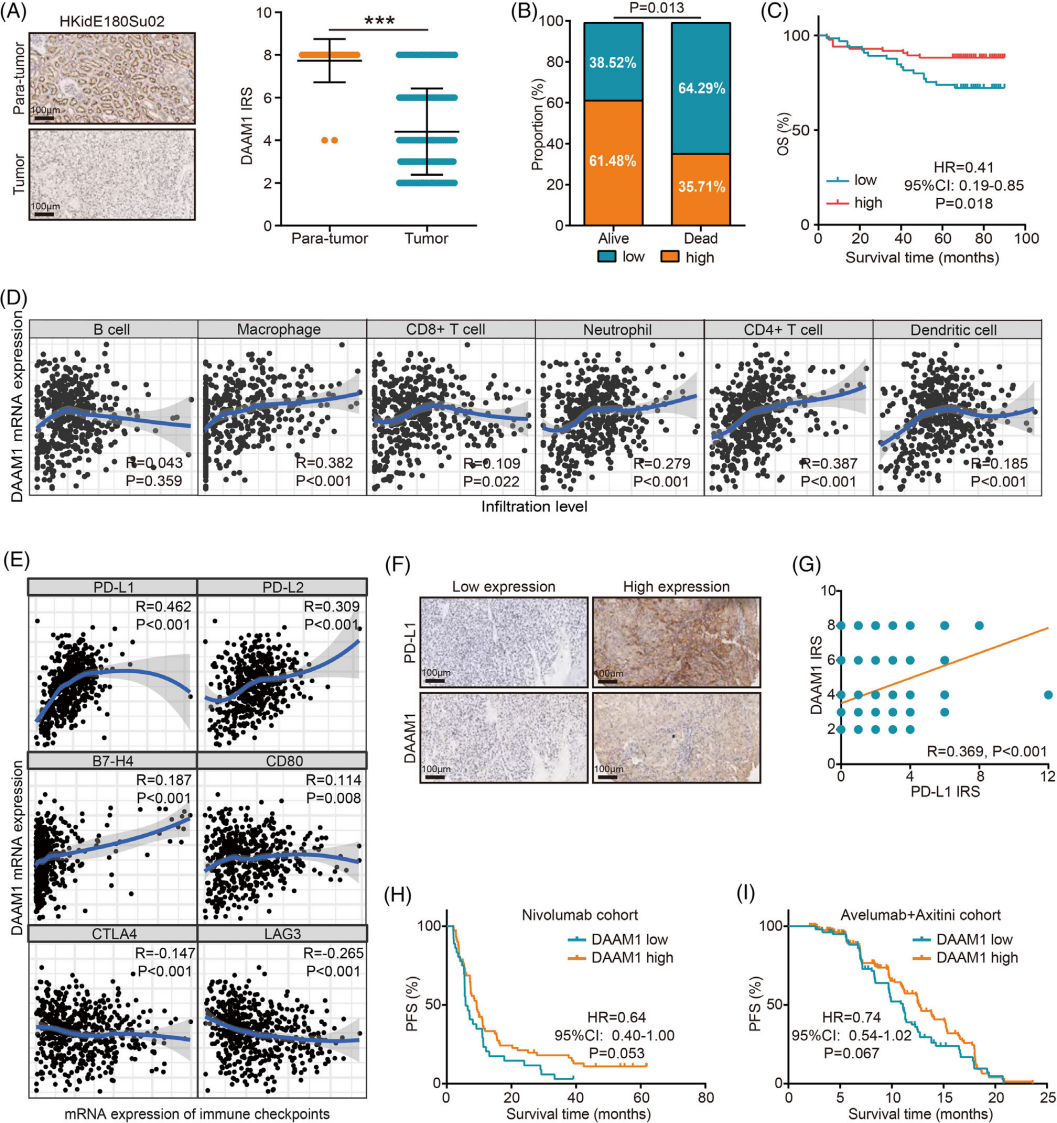
Expression, prognostic value, and immuno‐correlation of Disheveled‐associated activator of morphogenesis 1 (DAAM1) in kidney renal clear cell carcinoma (KIRC). (A) Representative immunohistochemistry (IHC) staining revealing DAAM1 expression in tumor and para‐tumor tissues and semiquantitative analysis of DAAM1 expression in the HKidE180Su02 cohort. Magnification, 200×. A total of 150 tumor and 30 para‐tumor samples were included for analysis. The immunoreactivity score (IRS) equals to the percentages of positive cells multiplied with staining intensity. The percentage of positively stained cells was scored as 0–4: 0 (<5%), 1 (6%–25%), 2 (26%–50%), 3 (51%–75%), and 4 (>75%). The staining intensity was scored as 0–3: 0 (negative), 1 (weak), 2 (moderate), and 3 (strong). (B) Association between DAAM1 expression and survival status in KIRC patients. (C) Association between DAAM1 expression and overall survival in KIRC patients. DAAM1 low expression, *n* = 65; DAAM1 high expression, *n* = 85. (D) The correlation between DAAM1 and the levels of B cell, CD8^+^ T cell, CD4^+^ T cell, macrophage, neutrophil, and dendritic cell. (E) Correlation analysis between DAAM1 and several immune checkpoints expressions. (F) Representative IHC staining revealing DAAM1 and programmed cell death 1 ligand 1 (PD‐L1) expressions in tumor tissues. (G) Correlation between DAAM1 and PD‐L1 expressions in the HKidE180Su02 cohort. (H and I) Association between DAAM1 expression and progression‐free survival in KIRC patients receiving Nivolumab or Avelumab + Axitini therapy. (H) DAAM1 low expression, *n* = 36; DAAM1 high expression, *n* = 71. (I) DAAM1 low expression, *n* = 104; DAAM1 high expression, *n* = 206.

Given the abnormal prognostic value, we next investigated the biological role of DAAM1 in KIRC. Based on the Linked Omics database, we screened the correlated genes and then performed enrichment analysis (Figure S4A–C). The results showed that DAAM1 was positively correlated with multiple oncogenic pathways in terms of KEGG and WiKiPathway enrichments, such as Wnt signaling pathway and transforming growth factor (TGF)‐β signaling pathway, indicating that DAAM1 was an oncogene in KIRC (Figure S4D,E). We also knockdown DAAM1 in 786‐O cells using siRNA (5ʹ‐TTAGATTGAGAACACTGGG‐3ʹ) and measured the proliferation and migration capacities by Cell Counting Kit‐8 (CCK‐8) and transwell assays (Figure S4F), and the results exhibited that DAAM1 knockdown notably inhibited cell proliferation and migration (Figure S4G,H). Taken together, the function of DAAM1 seemed to still oncogenic in KIRC.

It was curious that oncogenic DAAM1 was related to well prognosis in KIRC. We checked the correlations between DAAM1 expression and tumor‐infiltrating lymphocytes in KIRC and the results showed that DAAM1 was positively correlated with the levels of CD8^+^ T cell, CD4^+^ T cell, macrophage, neutrophil, and dendritic cell (Figure 1D). In addition, the expression of inhibitory immune checkpoints, such as PD‐L1, was adaptably upregulated in immuno‐hot tumors. Thus, we also examined the correlations between DAAM1 and immune checkpoints expressions, and we found that DAAM1 was tightly correlated with PD‐L1 and PD‐L2 expressions among common immune checkpoints (Figure 1E). Furthermore, the positive correlation between DAAM1 and PD‐L1 was also verified in the HKidE180Su02 cohort (Figure 1F,G). Although high immune infiltration in KIRC seems to been associated with poor prognosis, the correlations between specific immune cells infiltration and prognosis in KIRC are heterogeneous. Several immune cell types are still associated with better prognosis, such as CD19^+^ B lymphocytes.^5^ We currently did not know the significance of the correlation between DAAM1 and immune cells in KIRC. Thus, further studies are needed to be conducted based on this evidence. Next, we also investigated whether DAAM1 was a biomarker for PD‐1/PD‐L1 blockade in KIRC. The results from public cohorts revealed that high DAAM1 expression was related to well prognosis in the KIRC patients receiving Nivolumab^6^ or Avelumab + Axitini^7^ therapy (Figure 1H,I). It should be noted that the prognosis analysis shows no statistically significant differences but a trend. We also used the PRJEB23709, GSE176307, and IMvigor210 cohorts to check the prognostic value of DAAM1 in melanoma and urothelium cancer, and the results showed that DAAM1 could not predict prognosis in melanoma and urothelium cancer patients (Figure S5A–C). Overall, these data supported that DAAM1 was a novel and unique biomarker for immune cells infiltration and PD‐1/PD‐L1 blockade therapy in KIRC.

In the past decade, the roles of Formin protein in human cancers were well studied.^8^ As a member of Formin family, the oncogenic functions of DAAM1 in multiple cancers have been preliminarily explored. DAAM1 acts as a classical mediator in the Wnt/planar cell polarity (PCP) pathway by forming a complex with Disheveled protein and then activating Rho protein, leading to cytoskeletal remodeling and cell migration.^8^ However, the non‐classical role of DAAM1 has not been observed. In this research, we report that DAAM1 is downregulated in tumor tissues and predicts well prognosis. In addition, DAAM1 is positively correlated with PD‐L1 expression and as a novel biomarker for PD‐1/PD‐L1 blockade in KIRC. Although PD‐L1 is the most commonly used biomarker, its predictive efficiency may be affected due to the presence of modifications such as glycosylation.^9^ Thus, alternative biomarkers remain crucial in clinical practice. For a long time, researchers have habitually associated Formin proteins with cytoskeletal rearrangement. An interesting research revealed that formin homology 2 domain containing 1 (FHOD1) and formin‐like 1 (FMNL1), family members of Formin, are correlated with tumor‐infiltrating lymphocytes in gastric cancer, which first linked Formin proteins with anti‐tumor immunity.^10^ However, molecular mechanisms underly Formin‐mediated anti‐tumor immunity have not been investigated, which is also a major shortcoming in the current research. However, the current research provides DAAM1 as a novel biomarker for PD‐1/PD‐L1 blockade in KIRC.

## AUTHOR CONTRIBUTIONS

Y.Z. and J.X. conceived the study and participated in the study design, performance, coordination, and project supervision. J.M., H.F., J.Z., and D.H. collected the public data and performed the bioinformatics analysis. J.M., H.F., and J.Z. performed the immunohistochemistry staining. Y.Z. and J.X. revised the manuscript. Y.Z. and J.X. got financial supports. All authors reviewed and approved the final manuscript.

## CONFLICT OF INTEREST

The authors declare they have no conflicts of interest.

## ETHICS STATEMENT

Ethical approval for the study of tissue microarray slide was granted by the Clinical Research Ethics Committee, Outdo Biotech (Shanghai, China).

## Supporting information

Supp Information

## Data Availability

All data supported the results in this study are showed in this published article and its supplementary files. Besides, original data for bioinformatics analysis could be downloaded from corresponding platforms.
