# Supplementary material for: Pan‐cancer analysis revealing DAAM1 as a novel predictive biomarker for PD‐1/PD‐L1 blockade in clear cell renal cell carcinoma
Source: MedComm (2020). 2022 Oct 27;3(4):e177. doi: 10.1002/mco2.177 (PMC9609439; doi:10.1002/mco2.177)
Supplement: Supplementary file 1 — Supp Information [file MCO2-3-e177-s001.docx]

**Supplementary Files**

**Title:** Pan-cancer analysis revealing DAAM1 as a novel predictive biomarker for PD-1/PD-L1 blockade in clear cell renal cell carcinoma

**Running Title:** Novel predictive biomarker DAAM1 in KIRC

**Author list:** Jie Mei ^1,*^, Honghong Fan ^1,*^, Jiaofeng Zhou ^2,*^, Dingwei Huang ^2^, Junying Xu ^1,#^, Yichao Zhu ^2,#^

^⁎^ Equal contribution

^#^ Correspondence to: Yichao Zhu and Junying Xu

**Authors’ affiliations:**

1. Department of Oncology, The Affiliated Wuxi People's Hospital of Nanjing Medical University, Wuxi, 214023, Jiangsu, China.
2. Department of Physiology, Nanjing Medical University, Nanjing, 211166, China.

**Supplementary Methods and** **Materials**

**Public data collection**

We downloaded the unified and standardized pan-cancer dataset TCGA-TARGET-GTEx (PANCAN) dataset from the UCSC (https://xenabrowser.net/) database, and further we extracted the RNA-sequencing data of DAAM1 in each sample, which was log_2_(x+0.001) transformed, and finally the expression data and survival information of common solid tumors was remained for further analysis. In addition, RNA-sequencing of DAAM1 and survival information of the TCGA-KIRC dataset (IlluminaHiSeq) and KIRC patients receiving Nivolumab ^1^ or Avelumab+Axitini ^2^ were also obtained. Moreover, the GSE176307 ^3^ dataset was downloaded from the Gene Expression Omnibus (GEO, <http://www.ncbi.nlm.nih.gov/geo/>) database and the PRJEB23709 ^4^ dataset was downloaded from the Tumor Immune Dysfunction and Exclusion (TIDE, http://tide.dfci.harvard.edu/) database. The expression and clinical data of the IMvigor210 cohort ^5^ were obtained from the corresponding website (<http://research-pub.gene.com/IMvigor210CoreBiologies/>). For survival analysis, samples with a follow-up time of less than 60 days or two months were excluded.

**LinkedOmics database analysis**

Linked Omics (http://www.linkedomics.org/login.php) is a web-based tool used to handle the TCGA data ^6^. In this research, the Linked Omics was used to perform enrichment analysis of DAAM1 in KIRC in terms of KEGG and WiKipathway. For all parameters, the default choices were utilized.

**Cell culture and siRNA transfection**

KIRC cell line 786-O was purchased from KeyGEN (Nanjing, China) and cultured in corresponding RPMI-1640 media and added with 10% fetal bovine serum (FBS) at 37 °C with 5% CO_2_. All experiments were performed with mycoplasma-free cells. For siRNA transfection, 786-O cells were transfected with DAAM1siRNA (5′-TTAGATTGAGAACACTGGG-3′) ^7^ or control siRNA using Lipofectamine 3000 Reagent (Cat. L3000015, Invitrogen, CA).

**Western blotting analysis**

786-O cells were plated in maintained in 6-well plates. Total proteins of cells were harvested using lysis buffer. Then, SDS-PAGE and Western blotting analysis were conducted referring to standardized protocols. The primary antibodies used as follows: DAAM1 (1:1000 dilution, Cat. 14876-1-AP, ProteinTech) and Tubulin (1:2000 dilution, Cat. 11224-1-AP, ProteinTech). DAAM1 protein levels were standardized to Tubulin.

**Cell proliferation and migration assay**

For cell proliferation assay, 786-O cells were digested using 0.25% trypsin for 2 minutes and resuspended with DMEM media containing 10% FBS. Suspended cells were seeded on a 96-well plate with the cell density adjusted to 1×10^4^ cells/ml (100 μl/well) and maintained at 37°C for 24, 48, and 72 h, respectively. After which 10 μl CCK-8 reagent was injected into each well, then, the plate was maintained in the incubator for 1 hour. The OD value of each well was examined at 450 nm using a microplate reader. In addition, the capacities of cell migration were analyzed by Boyden chamber assay. Briefly, 1×10^5^ 786-O cells (200 μl) in serum-free medium were inoculated to the upper side of the Boyden chamber (8.0-μm, Cat. 3422, Corning, NY, USA), and media containing 20% FBS (500μl) was added to the lower side. After 48 hours, the migratory cells on the lower side were fixed with 4% paraformaldehyde and stained with 0.2% crystal violet. To quantify the percentage of stained cells, cells were counted from three random microscopic fields at 200×.

**TIMER database analysis**

TIMER analysis (http://www.cistrome.dfci.harvard.edu/TIMER/) was developed to assess immune cell infiltration in data from the TCGA dataset ^8^. We investigated the correlations between DAAM1 and the abundance of immune cells, including B cell, CD4+ T cell, CD8+ T cell, neutrophil, macrophage, and dendritic cell. In addition, the correlations between DAAM1 and immune checkpoints expressions were also checked using the TIMER database. The scatterplots were displayed, showing Spearman’s correlation and statistical significance.

**Collection of KIRC specimens**

Three independent KIRC (Cat. HKidCRC060CS01, HKidCRC150CS01, and HKidE180Su02) tumor tissue microarrays (TMAs) were purchased from Outdo BioTech (Shanghai, China). The HKidCRC060CS01 cohort contained 30 tumor and para-tumor samples, the HKidCRC150CS01 cohort contained 120 tumor and 30 para-tumor samples, and the HKidE180Su02 cohort contained 150 tumor and 30 para-tumor samples. Detailed clinic-pathological information of three TMAs and follow-up data for the HKidE180Su02 cohort were provided by Outdo BioTech. Ethical approval was granted by the Clinical Research Ethics Committee in Outdo Biotech (Shanghai, China).

**IHC staining and semi-quantitative assessment**

Immunohistochemistry (IHC) staining was conducted on the above sections according to the standardized procedures. The primary antibodies used were as follows: anti-DAAM1 (1:500 dilution, Cat. 14876-1-AP, ProteinTech) and anti-PD-L1 (Ready-to-use, Cat. GT2280, GeneTech). Antibody staining was visualized with DAB and hematoxylin counterstain, and stained sections were captured using Aperio Digital Pathology Slide Scanners. The stained sections were independently evaluated by two pathologists. Expression levels of DAAM1 and PD-L1 in tumor cells were semi-quantitatively assessed by estimating the immunoreactivity score (IRS) ^9^. Specifically, the IRS equals to the percentages of positive cells multiplied with staining intensity. The percentage of positively stained cells was scored as 0–4: 0 (< 5%), 1 (6–25%), 2 (26–50%), 3 (51–75%) and 4 (> 75%). The staining intensity was scored as 0–3: 0 (negative), 1 (weak), 2 (moderate), and 3 (strong)

**Statistical analysis**

All statistical analyses were conducted using SPSS 26.0 and R 4.0.2. All data are presented as means ± SDs. The difference between the two groups was analyzed by Student’s t-test or Mann Whitney test. Survival analysis was performed by log-rank test and Cox regression analysis. Correlation analysis between two variables was analyzed by Pearson test. All statistical tests were two-sided, and P value ≤ 0.05 was considered statistically significant.

**References**

1. Braun DA, Hou Y, Bakouny Z, et al. Interplay of somatic alterations and immune infiltration modulates response to PD-1 blockade in advanced clear cell renal cell carcinoma. *Nature medicine.* 2020;26(6):909-918.

2. Motzer RJ, Robbins PB, Powles T, et al. Avelumab plus axitinib versus sunitinib in advanced renal cell carcinoma: biomarker analysis of the phase 3 JAVELIN Renal 101 trial. *Nature medicine.* 2020;26(11):1733-1741.

3. Rose TL, Weir WH, Mayhew GM, et al. Fibroblast growth factor receptor 3 alterations and response to immune checkpoint inhibition in metastatic urothelial cancer: a real world experience. *Br J Cancer.* 2021;125(9):1251-1260.

4. Gide TN, Quek C, Menzies AM, et al. Distinct Immune Cell Populations Define Response to Anti-PD-1 Monotherapy and Anti-PD-1/Anti-CTLA-4 Combined Therapy. *Cancer Cell.* 2019;35(2):238-255 e236.

5. Necchi A, Joseph RW, Loriot Y, et al. Atezolizumab in platinum-treated locally advanced or metastatic urothelial carcinoma: post-progression outcomes from the phase II IMvigor210 study. *Ann Oncol.* 2017;28(12):3044-3050.

6. Vasaikar SV, Straub P, Wang J, Zhang B. LinkedOmics: analyzing multi-omics data within and across 32 cancer types. *Nucleic acids research.* 2018;46(D1):D956-d963.

7. Rodriguez-Hernandez I, Maiques O, Kohlhammer L, et al. WNT11-FZD7-DAAM1 signalling supports tumour initiating abilities and melanoma amoeboid invasion. *Nat Commun.* 2020;11(1):5315.

8. Li T, Fan J, Wang B, et al. TIMER: A Web Server for Comprehensive Analysis of Tumor-Infiltrating Immune Cells. *Cancer Res.* 2017;77(21):e108-e110.

9. Mei J, Liu Y, Yu X, et al. YWHAZ interacts with DAAM1 to promote cell migration in breast cancer. *Cell Death Discov.* 2021;7(1):221.

**Supplementary Table**

Table S1. Univariate and multivariate survival analysis in patients with KIRC.

| Clinic-pathological parameters | | Univariate analysis | | | Multivariate analysis | | |
| --- | --- | --- | --- | --- | --- | --- | --- |
|  |  | HR | 95%CI | P value | HR | 95%CI | P value |
| Gender | male vs. female | 2.59 | 0.90-7.48 | 0.078 |  |  |  |
| Age | >60 vs. 60 | 2.50 | 1.18-5.29 | **0.016** | 1.57 | 0.71-3.48 | 0.266 |
| Grade | moderate/poor vs. well | 2.47 | 1.00-6.10 | **0.049** | 1.26 | 0.41-3.86 | 0.692 |
| T stage | T2-3 vs. T1 | 8.35 | 3.93-17.72 | **<0.001** | 5.61 | 2.16-14.56 | **<0.001** |
| N stage | N1 vs. N0 | 23.49 | 6.29-87.76 | **<0.001** | 5.93 | 1.53-22.97 | **0.010** |
| TNM stage | 2-3 vs. 1 | 8.35 | 3.93-17.72 | **<0.001** |  |  |  |
| DAAM1 | high vs. low | 0.41 | 0.19-0.88 | **0.022** | 0.45 | 0.20-1.01 | 0.054 |

*Bold values indicate P* < *0.05.*

**Supplementary Figures**


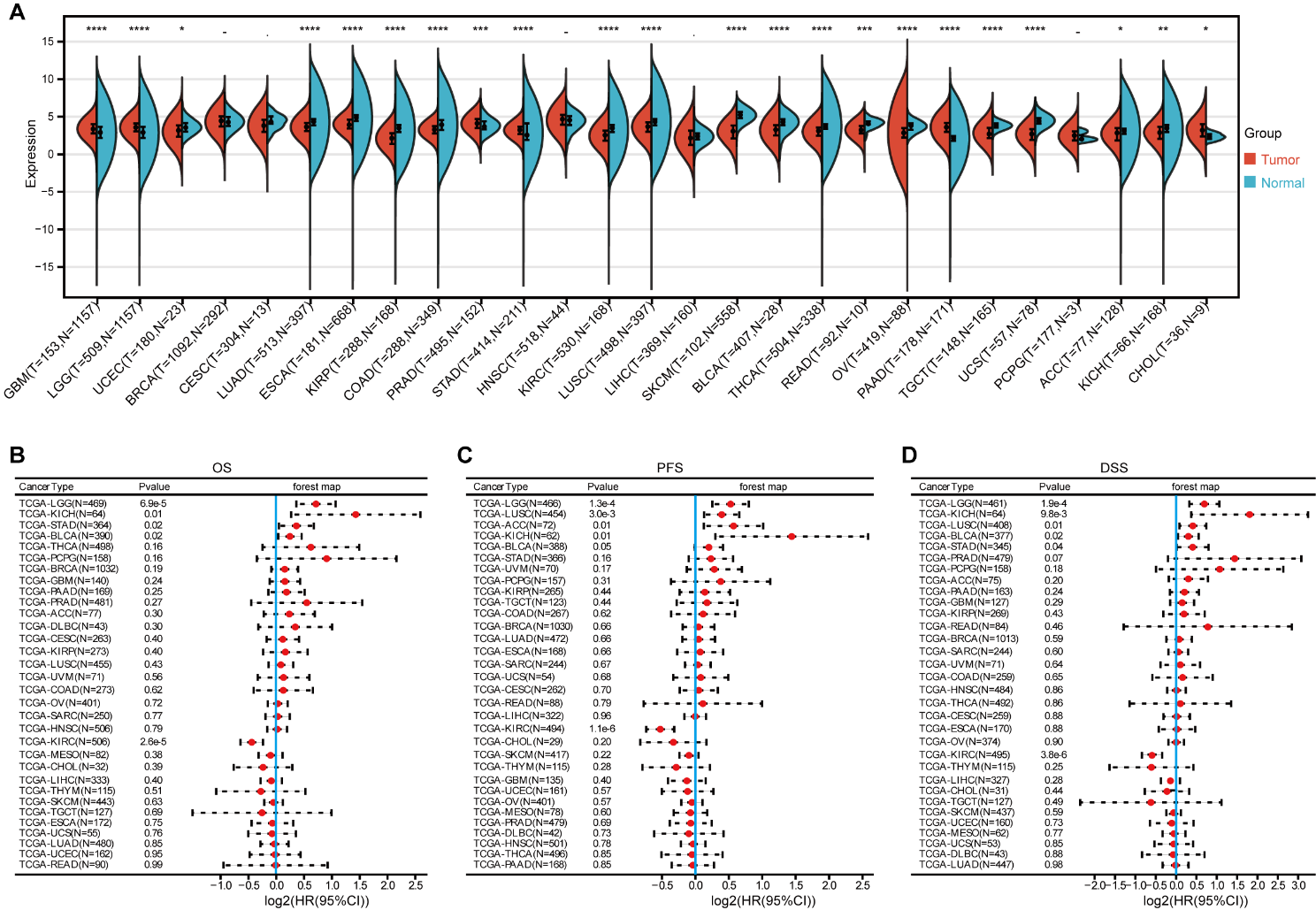


**Figure S1. Expressions and prognostic values of DAAM1 across cancer types.**

(A) Expression levels of DAAM1 in tumor and para-tumor tissues in various cancers. (B-D) Prognostic values of DAAM1 in various cancer in terms of overall survival, progression-free survival, and disease-specific survival.


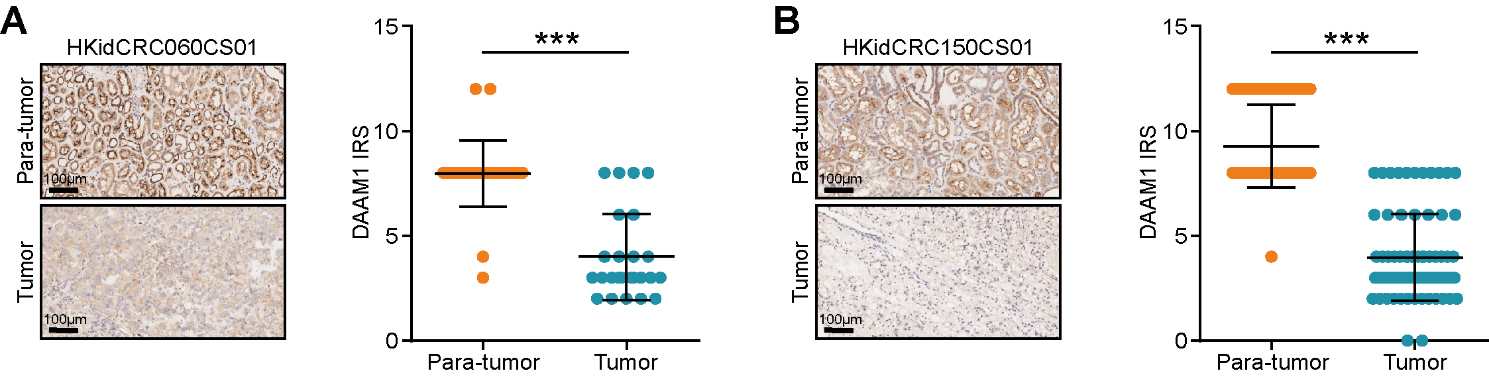


**Figure S2. Expression of DAAM1 in KIRC.**

(A) Representative images revealing DAAM1 expression in tumor and para-tumor tissues and semiquantitative analysis of DAAM1 expression in the HKidCRC060CS01 cohort. Magnification, 200×. A total of 120 tumor and 30 para-tumor samples were included for analysis. (B) Representative images revealing DAAM1 expression in tumor and para-tumor tissues and semiquantitative analysis of DAAM1 expression in the HKidCRC150CS01 cohort. Magnification, 200×. A total of 30 tumor and 30 para-tumor samples were included for analysis. IRS: immunoreactivity score. The IRS equals to the percentages of positive cells multiplied with staining intensity. The percentage of positively stained cells was scored as 0-4: 0 (< 5%), 1 (6-25%), 2 (26-50%), 3 (51-75%) and 4 (> 75%). The staining intensity was scored as 0-3: 0 (negative), 1 (weak), 2 (moderate), and 3 (strong).


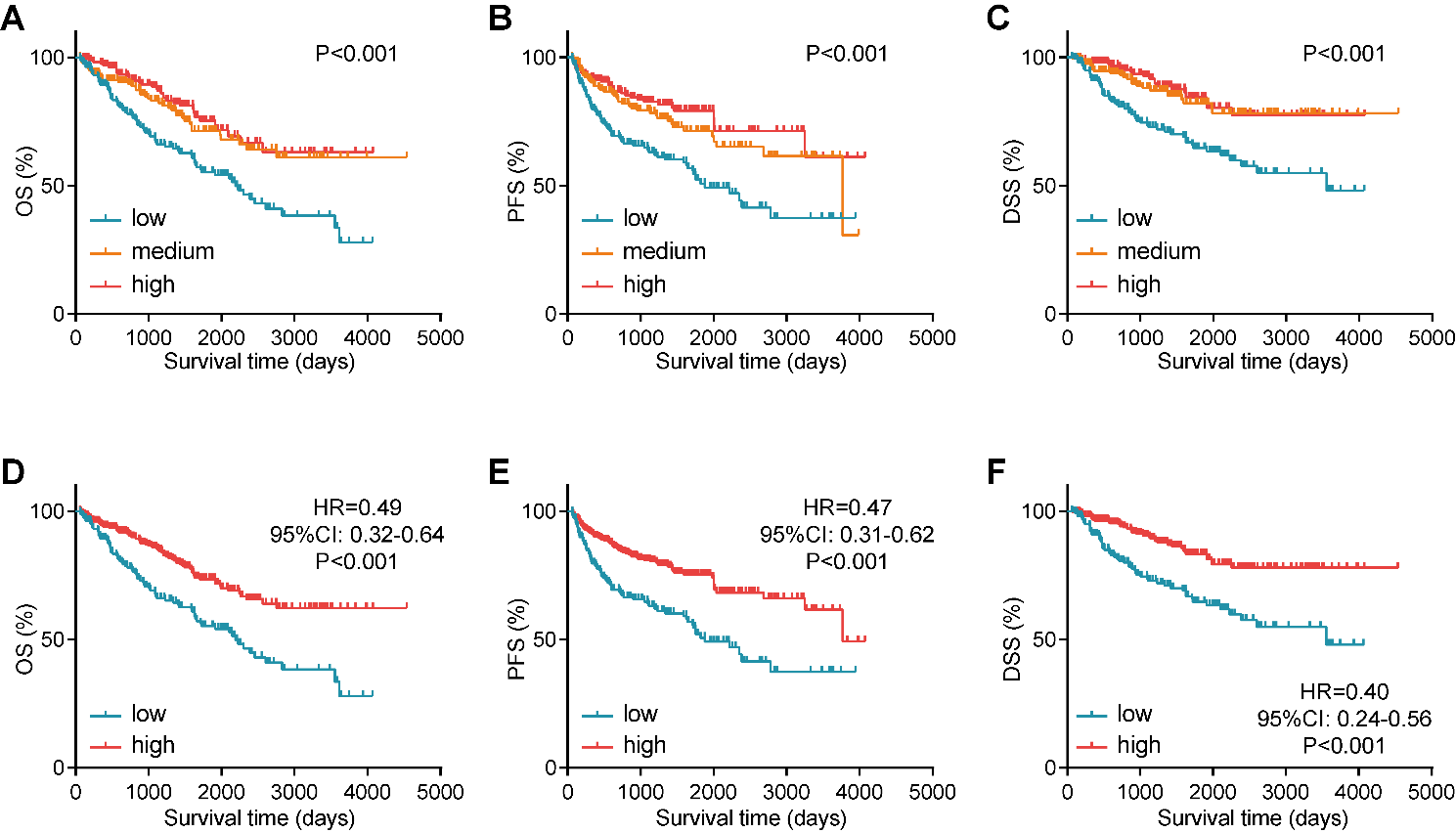


**Figure S3. Prognostic values of DAAM1 in KIRC.**

(A-C) Prognostic values of trisected DAAM1 expression in KIRC in terms of overall survival, progression-free survival, and disease-specific survival. DAAM1 low expression, n=162; medium expression, n=162, high expression, n=163. (D-F) Prognostic values of DAAM1 expression in KIRC in terms of overall survival, progression-free survival, and disease-specific survival. DAAM1 low expression, n=162; DAAM1 high expression, n=325.


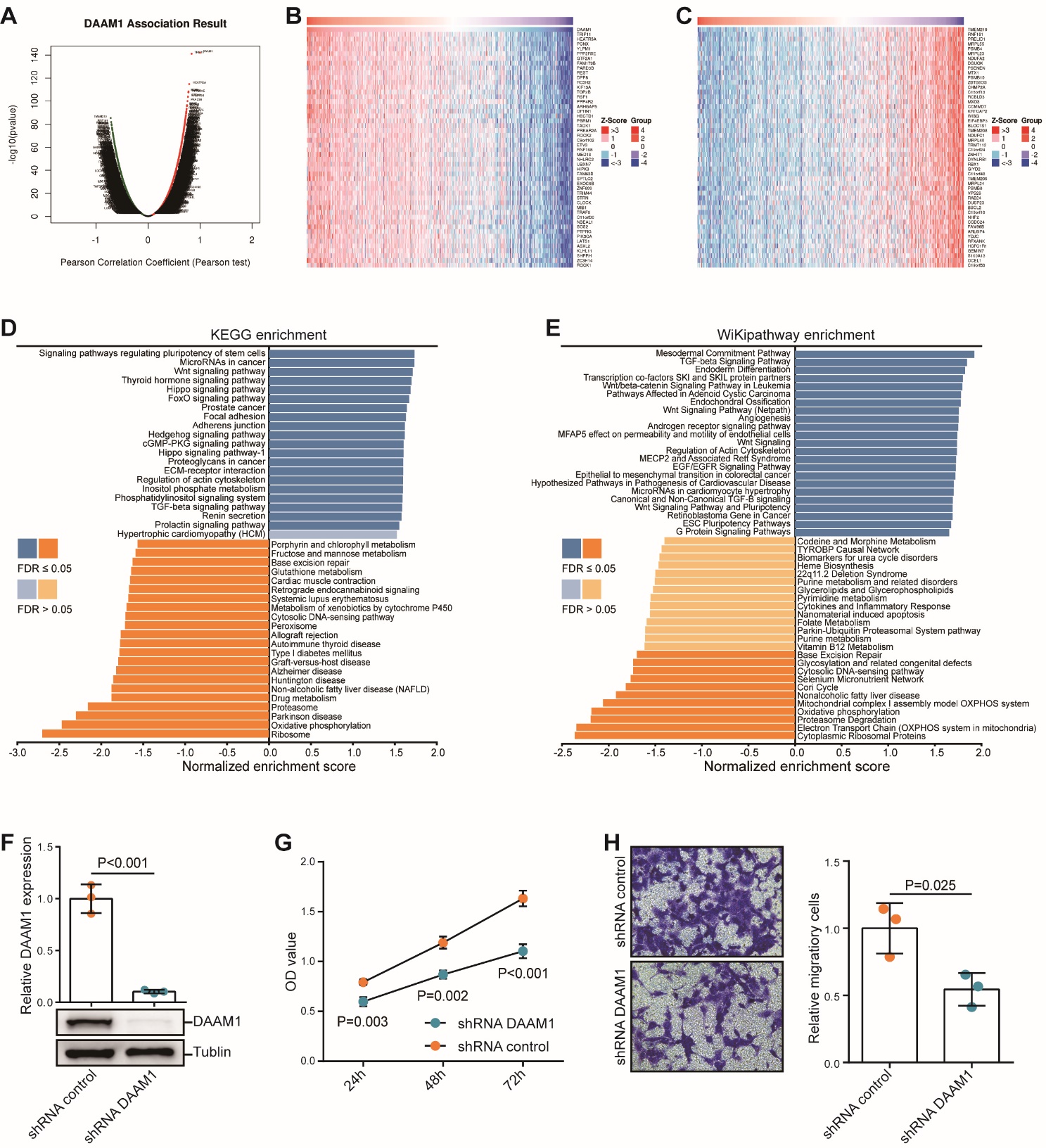


**Figure S4. Enrichment and biological analysis of DAAM1 in KIRC.**

(A) The global DAAM1 highly associated genes identified by the LinkedOmics database analysis in the TCGA-KIRC cohort. (B, C) Heat maps showing top 50 genes positively and negatively associated with DAAM1 in KIRC. (D, E) Significantly enriched KEGG and Wikipathway annotations of DAAM1 in KIRC. (F) The silencing efficiency of DAAM1 in 786-O cells was assessed by Western blotting. (G, H) The proliferative and migratory capacities of control and DAAM1-silencing 786-O cells was examined by CCK-8 and Boyden chamber assays.


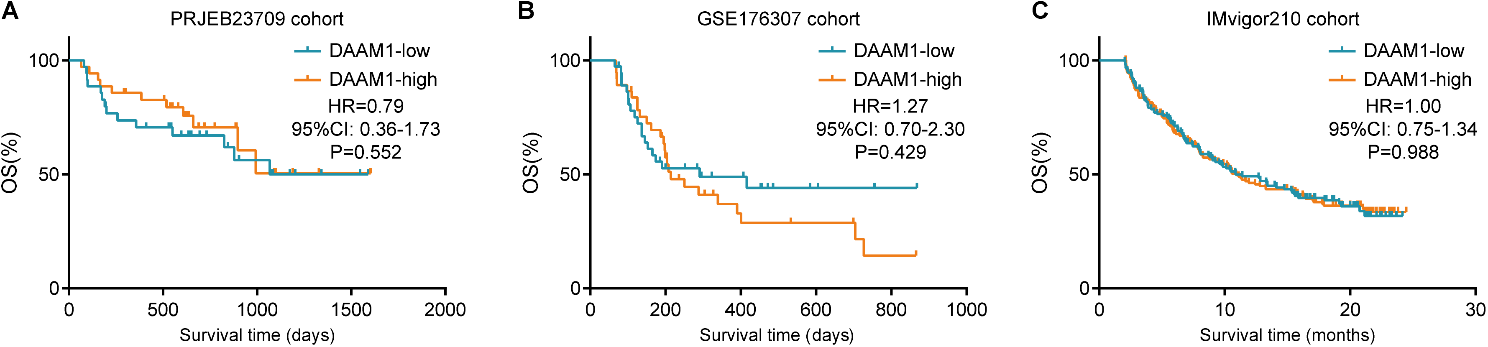


**Figure S5. Association between DAAM1 expression and prognosis in cancer patients receiving immunotherapy.**

(A) The PRJEB23709 cohort (melanoma cohort). DAAM1 low expression, n=35; DAAM1 high expression, n=35. (B) The GSE176307 cohort (urothelium cancer). DAAM1 low expression, n=37; DAAM1 high expression, n=37. (C) The IMvigor210 cohort (urothelium cancer). DAAM1 low expression, n=149; DAAM1 high expression, n=149.
